# Prospective outcomes following drainless superficial parotidectomy with sternocleidomastoid flap reconstruction

**DOI:** 10.1186/s40463-020-00472-z

**Published:** 2020-10-06

**Authors:** Jonathan C. Melong, Matthew H. Rigby, Martin Corsten, Jonathan R. B. Trites, Angela Bulter, S. Mark Taylor

**Affiliations:** Department of Otolaryngology Head & Neck Surgery, Dalhousie University, Halifax, Nova Scotia Canada

**Keywords:** Superficial parotidectomy, Drainless parotidectomy, Sternocleidomastoid flap, Frey’s syndrome, Contour defect, Facial paralysis

## Abstract

**Background:**

Patients undergoing superficial parotidectomy for benign parotid lesions are at risk of postoperative complications, most notably cosmetic complications such as facial paralysis and contour defects, and functional complications including Frey’s syndrome. Traditionally, surgical drains have been placed at the end of surgery to prevent hematoma and sialocele formation. However, this can increase the risk of postoperative complications and contribute to a prolonged course in hospital. To try and prevent these risks and complications, we introduced a novel technique of a drainless parotidectomy by reconstructing the resulting parotid bed defect with a superiorly based sternocleidomastoid (SCM) rotational flap and by placement of gelfoam into the wound bed and a facelift dressing postoperatively to provide additional hemostasis and avoid drain placement.

**Methods:**

All patients with benign parotid disease undergoing a drainless superficial parotidectomy and reconstruction with a superiorly based SCM rotational flap at our center were identified within a prospective cohort database between July 2010–2018. Primary outcomes included postoperative cosmetic and functional outcomes, complications and length of hospital stay. A secondary cost analysis was done to compare this novel technique to traditional superficial parotidectomy with surgical drain placement.

**Results:**

Fifty patients were identified within the database and were included in the final analysis. The average length of hospital stay was 1.02 days. All patients were satisfied with their aesthetic outcome at 1 year. During long term follow-up, 63% of patients reported normal appearance of the operated side. Seven patient’s (14%) developed temporary facial paresis following surgery. All patients had resultant normal facial function at follow-up in 1 year. No patients developed subjective Frey’s Syndrome. Two patients (4%) developed a postoperative sialocele requiring drainage and one patient (2%) developed a hematoma on extubation requiring evacuation and drain placement. Cost analysis demonstrated a cost savings of approximately $975 per person following surgery.

**Conclusion:**

In the current study, we introduced a novel approach of a drainless superficial parotidectomy using a superiorly based SCM flap, gelfoam and placement of a post-operative facelift dressing. This drainless approach was associated with good long-term cosmetic and functional outcomes with few postoperative complications. This new technique may also offer the potential for long-term savings to the health care system.

## Introduction

Superficial parotidectomy has remained the standard of care for the management of benign parotid masses, allowing for complete tumour resection while reducing the risk of recurrence [1–3]. However, it comes with a cost. Postoperatively, patients are at risk of cosmetic defects, most commonly contour defects of the resected area due to loss of tissue bulk and facial paralysis, and functional complications including Frey’s syndrome and first bite syndrome [4–6].

One strategy to help overcome these risks, that is becoming increasingly popular, is placement of a graft or flap between the resection bed and overlying skin. This adds tissue bulk to help prevent a contour deformity and also acts as a barrier between the severed parasympathetic nerve fibers of the parotid gland and the sympathetic nerve fibers of sweat glands in the overlying skin to prevent Frey’s syndrome. One of the most commonly used flaps for this purpose is the sternocleidomastoid (SCM) flap [7, 8]. Although initial studies demonstrated promising results, more recent studies have been conflicting necessitating the need for further studies to help clarify the role of local rotational flaps in preventing contour defects and Frey’s syndrome [9–11].

Traditionally, surgical drains have been inserted following superficial parotidectomy to reduce the risk of post-operative hematoma and sialocele formation. Although this was previously considered a benign intervention, increasing evidence has demonstrated that surgical drains may increase the risk of postoperative complications by contributing to retrograde infection, scarring, fistula formation and causing damage to surrounding structures by mechanical pressure [12, 13]. In the case of parotid surgery, this is particularly concerning as it may lead to delayed healing, worsen facial contour defects, and could theoretically increase the risk of facial paresis from mechanical pressure of the drain on exposed branches of the facial nerve. The use of surgical drains has also been previously demonstrated to increase the length of postoperative hospital stay, which has considerable patient and health care utilization costs and considerations [14, 15].

In the current study, we introduced a novel surgical approach to superficial parotidectomy that avoids drain placement. This approach involves reconstruction of the parotid bed defect with a superiorly based sternocleidomastoid (SCM) rotational flap and placement of gelfoam into the wound bed to add tissue bulk, separate severed parasympathetic nerve fibers from the overlying skin and to provide additional hemostasis. Following wound closure, a facelift dressing is placed post-operatively to provide continuous pressure to the wound bed to avoid drain placement. The purpose of this study was to evaluate the aesthetic and functional outcomes, and postoperative complications of this drainless approach compared to traditional surgical techniques.

## Methods

This was a prospective, cohort study based on a database monitoring all patients with benign parotid disease undergoing a drainless superficial parotidectomy and reconstruction with a superiorly based SCM rotational flap by the senior author of the study at the QEII Health Sciences Centre in Halifax, Nova Scotia between July 2010–2018. The collection of information within the database was approved by our institutional research ethics board. Demographic details were recorded for all patients. Operative records and pathology reports were reviewed to ensure appropriate indication and to verify surgical details.

Patients were followed according to current standards of practice at 1 and 12 months postoperatively. After patients were discharged from care, they were contacted to review long term outcomes and complications. During long term follow-up, patients were asked if they had any general concerns since surgery, if they had any noticeable residual facial weakness, were asked to rate their level of facial symmetry when comparing the operated side to the non-operated side on a scale of 0 to 10 (0 - Normal appearance, symmetrical to the opposite side and 10 - Severe asymmetry with obvious scar) and asked to rate symptoms of Frey’s syndrome on a scale of 0 to 10 (0 - No obvious sweating and 10 - Severe sweating interfering with quality of life).

Exclusion criteria included any patients with malignant parotid disease, patients requiring deep lobe resection, patients on anticoagulants or with known coagulopathies at the time of surgery, or any operations requiring more extensive surgery such as a neck dissection.

### Procedure

**Fig. 1:**
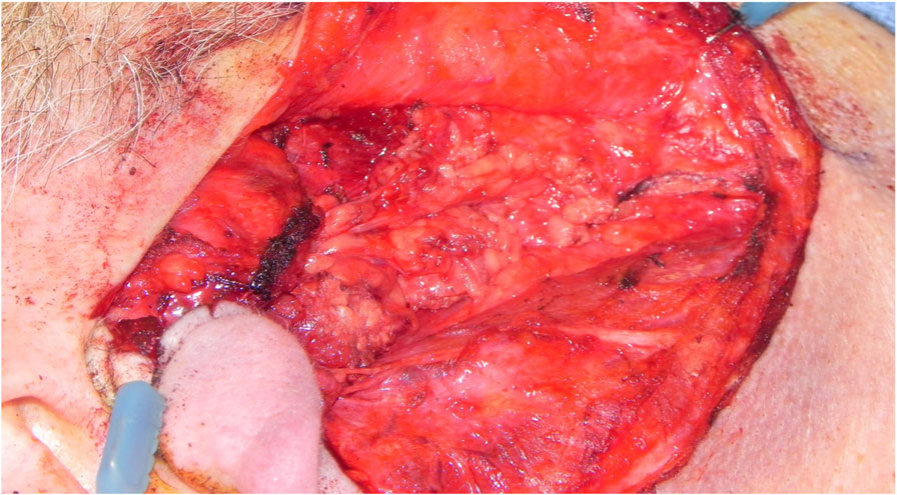
Superficial parotidectomy demonstrating exposed branches of the facial nerve

**Fig. 2:**
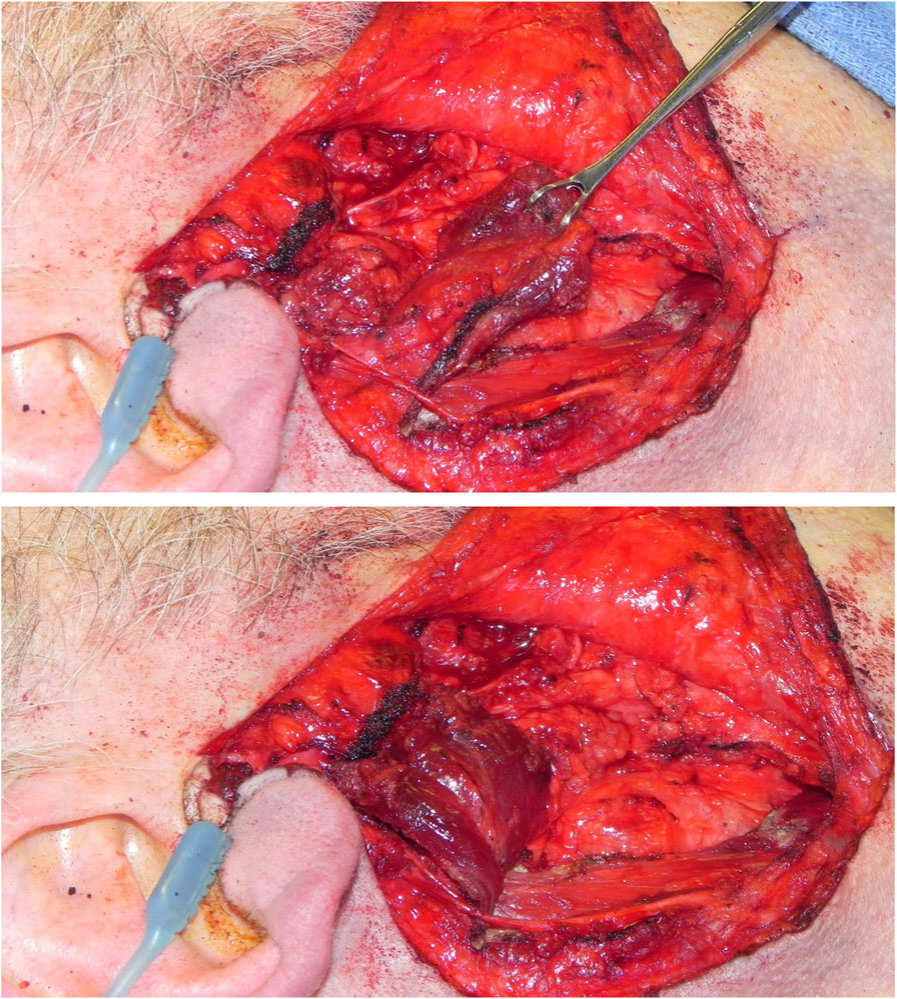
Superiorly based SCM flap elevated, rotated and sutured to the remaining SMAS in the parotid defect. A piece of gelfoam is placed between the muscle flap and parotid bed to provide additional hemostasis and bulk

**Fig. 3:**
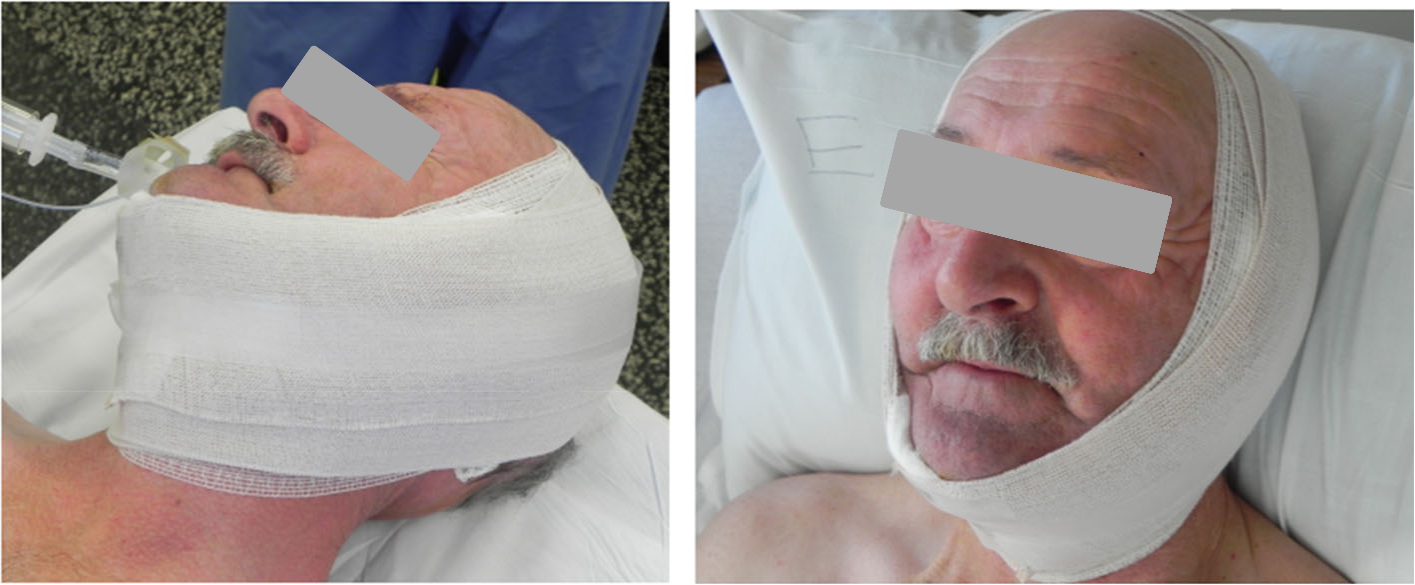
Facelift dressing placed post-operatively

A standard modified Blair incision was used to approach the parotid gland for the superficial parotidectomy. Facial nerve monitoring was used in all cases. The overlying skin flap was raised in a sub-superficial musculoaponeurotic system (SMAS) and sub-platysmal plane. A standard superficial parotidectomy was then performed with identification of the main trunk of the facial nerve and resection of the superficial parotid tissue in an anterograde fashion (Fig. 1). Following the superficial parotidectomy, the parotid contour deformity was repaired with a superiorly based SCM flap. The flap was designed from the anterior half of the superior portion of the SCM, based on the mastoid process, elevated, rotated and sutured to the remaining SMAS in the parotid defect (Fig. 2). The spinal accessory nerve was protected in all cases. A piece of gelfoam was placed between the muscle flap and the parotid bed for additional hemostasis and bulk. For wound closure, the deep layer was closed with an absorbable suture and the skin incision was closed with a non-absorbable monofilament suture. A standard facelift dressing was applied at the end of the case and removed the following day on postoperative day 1 (Fig. 3).

## Results

Between 2010 and 2018, 67 patients underwent a superficial parotidectomy by the senior author of the study for suspected benign parotid disease. Of these, 50 patients were included in the final analysis based on inclusion/exclusion criteria. Primary reasons for exclusion from the study included patients requiring deep lobe resection (*n* = 5), patients requiring drain insertion because of extensive resection (*n* = 8), patients who did not receive a SCM flap because of a minimal defect (*n* = 2) or high risk of recurrence (*n* = 1), and pathology demonstrating malignant disease (*n* = 1).

Of the 50 patients included in the study, 25 were male and 25 were female (Table 1). The mean age of the cohort at the time of surgery was 55.5 years. The mean time of follow-up according to routine clinical practice was 12.1 months. Thirty-three patients were contacted for long term follow-up. The mean time from surgery to long-term follow-up was 49 months. The most common diagnosis after final pathology review was pleomorphic adenoma (*n* = 25) followed by Warthin’s tumour (*n* = 18), basal cell adenoma (*n* = 4), onycocytoma (*n* = 2) and parotid cyst (*n* = 1).

**Table 1:** Patient Characteristics (*n* = 50)

Characteristic	Number of Patients (%)
Age, y	
Mean (Range)	55.5 (24–76)
Sex	
Male	25 (50%)
Female	25 (50%)
Pathology	
Pleomorphic Adenoma	25 (50%)
Warthin’s Tumour	18 (36%)
Basal Cell Carcinoma	4 (8%)
Onycocytoma	2 (4%)
Parotid Cyst	1 (2%)

### Hospital stay

The average length of hospital stay was 1.02 days. The majority of patients (98%) were discharged home the following day after surgery. Only one patient required an extra day in hospital because of the development of a hematoma on extubation requiring drain placement. The patient was discharged from hospital on postoperative day two, once drain output was below 30 mls over 24 h.

### Aesthetic and functional outcomes

All patients were satisfied with their aesthetic outcome at 1 year. There was no obvious visible contour defect or significant scarring as assessed by the senior author of the study. During long term follow-up, most patients reported maintenance of good facial contour and symmetry. Twenty one of thirty-three patients (63%) reported normal appearance of the operated side. Only six patients reported a visible contour defect. The overall mean score on the visual analogue scale was 0.89 out of 10.

Seven patients (14%) developed temporary postoperative facial paresis. Of these, six patients had mild weakness of the marginal mandibular branch and one patient had mild weakness of the temporal branch of the facial nerve. All patients had resultant normal facial function at follow-up in 1 year.

No patients in the cohort developed subjective Frey’s Syndrome either during follow-up at 1 year or during long term follow-up.

### Complications

Two patients (4%) developed a postoperative sialocele within the first week following surgery. In both cases, the sialocele was drained in clinic and patients were treated with antibiotics and pressure dressings on an outpatient basis. The sialocele ultimately resolved for both patients with conservative treatment with no need for further intervention and no long-term sequelae.

As mentioned previously, one patient (2%) developed a postoperative hematoma, which occurred during extubation. The incision site was re-opened and the hematoma was evacuated intraoperatively. No active sites of bleeding were identified. A drain was subsequently inserted and the incision site was closed with no further issues.

### Cost analysis

Previous studies have demonstrated that patients undergoing superficial parotidectomy with placement of a surgical drain remained in hospital on average between 2 and 3 days [14, 15]. Similar results were found at our own center, where a review of patients who had a drain placed following their superficial parotidectomy by the senior author of the study had an average postoperative hospital stay of 2.2 days prior to introduction of the current drainless surgical approach. We reviewed operating room times comparing the drainless surgical approach to traditional superficial parotidectomy with drain placement and found that on average, an extra 15 min was required to raise and inset the SCM flap. Taking this into consideration, we performed a cost analysis comparing patients undergoing traditional superficial parotidectomy with drain placement compared to the current cohort (Table 2). We found an average cost savings of approximately $975 per person following surgery.

**Table 2:** Cost analysis comparing drainless superficial parotidectomy with SCM flap, gelfoam and facelift dressing to traditional superficial parotidectomy with drain placement

Treatment Component ($)	Cost ($)
Superficial Parotidectomy with SCM Flap and Facelift Dressing	
Surgeon billing	1453.74
Anesthetist billing (166/h)	373.5
Nurse’s fee (37.62/h)	84.65
Operating room (300)	300
Gelfoam and facelift dressing	9.41
Hospital stay (1404/day)	1432.08
Total	3653.38
Traditional Superficial Parotidectomy with Drain Placement	
Surgeon billing	822.25
Anesthetist billing (166/h)	332
Nurse’s fee (37.62/h)	75.24
Operating room (300)	300
JP drain	10.40
Hospital stay (1404/day)	3088.80
Total	4628.69

## Discussion

While the most feared complication of parotid surgery is facial nerve paralysis, the most common complications affecting patient’s quality of life postoperatively are cosmetic defects and Frey’s syndrome [16, 17]. A proposed surgical intervention to overcome these risks is placement of a graft or flap between the resection bed and overlying skin to add tissue bulk and create a barrier between the severed parasympathetic nerve fibers of the parotid gland and the sympathetic nerve fibers of sweat glands in the overlying skin to prevent Frey’s syndrome. Different surgical approaches have been attempted in the past including the use of free fat grafts, interposition grafts with fascia lata or superficial temporal fascia, and local flaps including SCM, SMAS, and platysmal flaps [18–20]. The SCM flap is a particularly attractive option given its close proximity, robustness and low risk of complications [11, 21].

In the current study, we introduced a novel technique to reduce the risk of post-operative complications following superficial parotidectomy by rotation and inset of a superiorly based SCM flap, gelfoam and placement of a face-lift dressing at the completion of surgery to avoid surgical drains. We demonstrated that the majority of patients had excellent cosmetic results at one-year follow-up, which was maintained long term (mean 49 months) in the majority of patients. This is one of the few studies demonstrating long-term aesthetic outcomes with SCM flaps; highlighting the robustness of the flap.

Only seven (14%) patients in the current study developed temporary facial paralysis. This is in the lower range compared to rates previously demonstrated in the literature, where temporary facial paralysis following superficial parotidectomy has been found to range between 10 and 80% [4, 22, 23]. One possible explanation for this is that in most studies, a surgical drain was placed at the end of surgery. Theoretically, this may have increased the risk of temporary facial paralysis due to increased mechanical pressure placed on exposed facial nerve fibers. To avoid this complication, we not only avoided surgical drain placement, but also used the SCM flap and gelfoam to cover exposed branches of the facial nerve. Although theoretical, this may in part explain the low incidence of temporary facial paralysis seen in the current study.

Frey’s syndrome is a well described phenomenon following parotid surgery [24, 25]. In fact, previous studies have demonstrated that 40–95% of patients will have objective findings of Frey’s Syndrome following superficial parotidectomy [26–28]. However, only 10–25% of patients will have subjective symptoms [4, 26–28]. Given these findings, subjective as opposed to objective findings of Frey’s syndrome are arguably more important when assessing functional outcomes following parotid surgery. It has also been previously demonstrated that symptoms of Frey’s syndrome may not be present until 2–3 years after surgery, highlighting the importance of long-term follow-up and monitoring [28, 29]. Previous studies evaluating the efficacy of the SCM flap for preventing Frey’s syndrome have been conflicting [9–11]. This may, in part, be explained by small sample sizes and significant heterogeneity between studies with respect to surgical technique, assessment, and duration of follow-up. Despite these conflicting results however, a meta-analysis by Liu et al. [8] demonstrated that SCM flaps decreased both objective and subjective Frey’s syndrome following parotid surgery. In the current study, patients were explicitly asked if they had any symptoms of facial sweating when chewing or eating during their follow-up appointments. As all patients denied any symptoms of Frey’s Syndrome at 1 year and during long-term follow-up, no patients required further investigations or management.

With greater demands on the health care system, there has been increased emphasis on cost-effective health care delivery and utilization [30, 31]. Previous studies have demonstrated that one of the greatest expenses for surgical patients is length of time in hospital following surgery, which is prolonged in patients with surgical drains [14, 32, 33]. Given these findings, there has been increased interest in performing outpatient head and neck procedures, including parotid surgery [34–36]. A recent systematic review and meta-analysis by Flach et al. [37] demonstrated that outpatient parotidectomy appeared to be safe with no increased risk of postoperative complications or readmission rates compared to inpatient procedures. Most studies included in the final analysis however, evaluated outcomes for patients who were discharged from hospital with a surgical drain in place. Although this was associated with an overall cost savings, this approach would not be suitable for all patients. Furthermore, patients would still be at risk of complications associated with surgical drains as previously described [12, 13]. In the current study, we found an average cost savings of $975 per person, which was largely attributed to decreased length of hospital stay. This cost savings would vary slightly across centers based on individual center billing codes and funding plan arrangements. It should be noted that the authors of the current study are part of an alternate funding plan and there is no financial incentive to perform the SCM flap. In fact, given that there is no direct billing of procedures, anticipated cost savings would be even higher than demonstrated in the current study. Most importantly, however, this highlights that a drainless approach is a safe and cost-effective approach that avoids the need for outpatient drain management and its associated complications without sacrificing patient care.

This study is not without its limitations. One of the major limitations of the study is the inherent biases associated with small prospective, non-randomized controlled trials. To try and limit these biases, all patients in the study were asked to objectively assess their own cosmetic and functional outcomes following surgery on a scale from 0 to 10. This not only served to reduce assessor bias, but also provides valuable information on patient perceived outcomes following surgery. As mentioned previously, Frey’s syndrome was intentionally measured based on patient’s subjective symptoms as opposed to objective testing as previous studies have demonstrated that many patients will have objective findings of Frey’s syndrome in the absence of symptoms [26–28]. Despite these limitations, however, this study demonstrates promising results for the prevention of cosmetic and functional complications following superficial parotidectomy using a drainless surgical approach in combination with a SCM rotational flap.

## Conclusion

In the current study, we have introduced a novel drainless approach to superficial parotidectomy using a superiorly based SCM flap, gelfoam and a post-operative facelift dressing. This novel approach was associated with good long-term cosmetic and functional outcomes, few postoperative complications and has the potential to offer significant cost savings to the health care system.

## Data Availability

The datasets generated and/or analysed during the current study are available from the corresponding author on reasonable request.
